# Effects of bifid triple viable capsules dissolving at intestines combined with emergency comprehensive nursing on intestinal microorganism and nutritional status of patients with coronary heart disease after percutaneous coronary intervention

**DOI:** 10.3389/fmed.2025.1649671

**Published:** 2025-09-02

**Authors:** Ming Zuo, Zhi Liu

**Affiliations:** Emergency Department, Xuanwu Hospital, Capital Medical University, Beijing, China

**Keywords:** coronary heart disease, percutaneous coronary intervention, emergency comprehensive nursing, complications, bifid triple viable capsules dissolving at intestines, intestinal microorganism, nutritional status

## Abstract

**Objective:**

To evaluate the combined effects of bifid triple viable capsules dissolving at intestines and emergency comprehensive nursing on intestinal microbiota, cardiovascular function, and nutritional status in coronary heart disease (CHD) patients post-percutaneous coronary intervention (PCI).

**Study design:**

A randomized controlled trial.

**Place and duration of study:**

This study was conducted at Capital Medical University from March 2023 to January 2024.

**Methodology:**

A total of 110 CHD patients who underwent PCI were randomized into two groups: the control group (CG, *n* = 55) receiving routine emergency nursing, and the study group (SG, *n* = 55) receiving emergency comprehensive nursing combined with bifid triple viable capsules (0.63 g, twice daily). Key outcomes, including left ventricular ejection fraction (LVEF), blood pressure, length of hospital stay, psychological well-being, nursing satisfaction, intestinal microbiota composition, and nutritional and lipid profiles, were measured before and after 8 weeks of intervention.

**Results:**

The SG demonstrated significantly higher improvements in LVEF and blood pressure and shorter hospital stays compared to the CG (*p* < 0.05). Psychological well-being, self-efficacy, and nursing satisfaction scores were significantly better in the SG (*p* < 0.05). Additionally, the SG showed greater restoration of lactobacillus and bifidobacterium levels and reduction in escherichia coli levels (*p* < 0.05). Nutritional markers, including hemoglobin, albumin, transferrin, and prealbumin, as well as lipid profiles, such as TG, TC, LDL-C, and HDL-C, improved significantly in the SG compared to the CG (*p* < 0.05).

**Conclusion:**

The combination of bifid triple viable capsules and emergency comprehensive nursing significantly improved cardiovascular function, reduced complications, enhanced self-efficacy and quality of life, and optimized intestinal microbiota and nutritional status in CHD patients after PCI.

## Introduction

Coronary heart disease (CHD) belongs to a kind of heart disease that causes myocardial ischemia, hypoxia and even necrosis because of vascular cavity obstruction, stenosis and (or) coronary spasm caused by coronary artery (coronary artery) atherosclerosis (1). According to guidelines published by the European Society of Cardiology in 2019, CHD can be separated into acute coronary syndromes (ACS) as well as chronic coronary syndromes (CCS) (2). As one of the major cardiovascular diseases affecting the global population and human health, CHD is an important cause of death for residents both abroad and at home (3). The high incidence of CH D is middle-aged and elderly people, some patients may show no significant symptoms, and the typical symptoms of symptomatic patients are chest pain (often feel a sense of contraction and pressure in the precardiac area) (4). At the same time, patients may have nausea, respiratory depression, sweating and other systemic symptoms, with the prolonged course of CHD can cause heart failure, cardiogenic shock, arrhythmia and other complications, therefore, more attention should be paid to this disease (5).

Conservative drug therapy is the most common treatment for patients with CHD (6). Although this kind of drug therapy can achieve a certain maintenance treatment effect, the long-term use of such drugs may have certain side effects, and some severe patients may have acute heart failure due to long-term use, which restricts the efficacy of conservative drug treatment to a certain extent (7). With the progress of cardiac medicine research in recent years, percutaneous coronary intervention (PCI) has been widely applied in clinic (8). PCI means that doctors re-clear the narrow coronary artery cavity with the help of the ever-developing cardiac catheterization technology, so as to restore and promote the myocardial blood supply, which is the cornerstone of the treatment of CHD (9). With the development of science and technology along with the accumulation of experience, PCI technology develops rapidly, and the equipment is updated iteratively (10). The variety of stent selection and the expansion of surgical approaches have promoted the application of PCI in the treatment of CHD patients (11). Nevertheless, postoperative patients need long-term medication, and poor treatment compliance and improper health behavior will affect the prognosis (12). Therefore, auxiliary and effective nursing intervention is needed to promote the prognosis of patients.

Emergency comprehensive nursing intervention belongs to a novel nursing model, which can offer comprehensive and systematic nursing services for patients along with improve nursing quality (13). With the deepening of nursing research, the comprehensive nursing intervention model has been widely applied in clinical practice, and has achieved remarkable results (14).

Intestinal flora is known as the “human microbial organ,” which can effectively regulate the innate and acquired immunity of the body, and the disturbance of intestinal flora can induce lesions of multiple systems or organs (15). Studies have found that due to the large volume load of CHD patients, intestinal flora disturbance promotes the increase of intestinal permeability, accelerates the progression of CHD after excessive toxins enter the blood circulation, and the worse the heart function, the faster the spread of pathogenic bacteria (16). Recent studies have shown that intestinal microbial imbalance can cause adverse cardiovascular events after CHD (17).

Bifid triple viable capsules dissolving at intestines is a new type of microecological preparation, the components of which are: bifidobacterium longiformis, *lactobacillus acidophilus* and enterococcus faecalis, which can effectively adjust the balance of intestinal flora, inhibit and remove pathogenic bacteria in the intestine, reduce the production of enterotoxin, promote the body’s digestion of nutrients, synthesize vitamins required by the body, stimulate the body’s immunity (18). As reported previously, probiotics can improve lipid profiles in CHD patients (19) and effectively prevents myocardial injury from CHD patients (20). However, the role of bifid triple viable capsules in CHD patients after PCI remains unclear.

In this study, we aimed to explore the impacts of bifid triple viable capsules dissolving at intestines combined with emergency comprehensive nursing on intestinal microorganism and nutritional status of CHD patients after PCI.

## Data and methods

### General data

One hundred and ten CHD patients who underwent elective PCI in our hospital from March 2023 to January 2024 were selected, followed by randomly dividing into control group (CG, *n* = 55) and study group (SG, *n* = 55). The CG contained 30 males and 25 females, aged 49–75 years, with a mean age of (61.73 ± 8.72) years old. The duration of CHD was (5.74 ± 0.92) years, ranging 2–8 years. The SG contained 29 males and 26 females, aged 50–76 years, with a mean age of (61.78 ± 8.75) years old. The duration of CHD was (5.72 ± 0.89) years, ranging 2–9 years. No difference was discovered in general data between 2 groups (*p* > 0.05). Inclusion criteria: (1) Patients with symptoms of angina pectoris were diagnosed as CHD by coronary angiography, which met the diagnostic criteria for CHD of the American College of Cardiology/American Heart Association 2007; (2) Clear awareness, understanding and communication skills; (4) No other serious diseases; Exclusion criteria: (1) Severe coronary artery lesions, intravascular diffuse sclerosis and calcification, requiring coronary artery bypass grafting; (2) Patients with brain, kidney, lung and other organic diseases; (3) Patients with mental illness. This study was approved by the ethics committee of Capital Medical University on February, 3, 2023, and the approval number was KS2022174-1.

### Randomization

A group randomization design was adopted for random grouping. The random allocation sequence was generated by a computer. The allocation confidentiality measures were achieved through sequential numbering, sealing, and opaque envelopes. After being deemed to meet the inclusion criteria, patients were randomly assigned to the CG or the SG in a 1:1 ratio.

### Treatments

The CG was given healthy lifestyle guidance, such as smoking and alcohol cessation, a low salt and fat diet, and appropriate exercise. The control group was treated with conventional drugs such as statins, antiplatelet, nitrates, calcium channel antagonists and β-blockers.

On the basis of the CG, the SG was treated orally with bifid triple viable capsules dissolving at intestines (Manufaturer: Jincheng Haelsth Pharmaceutical Co., LTD., Specification: 0.21 g), 0.63 g/time, 2 times/d.

Both groups were treated for 8 weeks.

## Methods

The CG adopted emergency routine nursing, vital signs were monitored, oxygen inhalation support, water and electrolyte correction intervention were given to the patients, and the patients’ condition was evaluated and PCI was performed actively with the doctors. The precautions of self-management should be emphasized to patients after PCI.

The SG was given emergency comprehensive nursing, and the specific contents were as follows:

1. Preoperative nursing. On the basis of routine monitoring and life support treatment, the implementation of nursing included: (a) Psychological intervention: at the reception, the nurse warmly received the patient and his family, asked the patient’s situation with a kind tone and patience, carefully cared for the patient and fully understood the patient’s physical and mental feelings, and comforted the patient and his family emotionally. Regardless of the outcome of the prognosis assessment of the patient, the patient and his family were given positive assurance and encouragement. Nurses introduced the excellent qualifications and rich experience of PCI medical team to patients, introduced successful cases, and conveyed positive information to patients. (b) Disease education: Through one-to-one explanation, patients could understand the treatability of acute myocardial infarction, the maturity of PCI, the principle and advantage of PCI. Nurses informed the patient of possible adverse reactions during PCI, and informed the corresponding treatment methods and matters that the patient needed to cooperate with. (c) Drug intervention: Before surgery, nurses asked the patient and his family whether they had a history of drug use and the specific name and amount of drugs, and then informed the medical staff and prescribe drugs.
2. Intraoperative nursing. Based on the implementation of routine intraoperative monitoring, the implementation of nursing includes: (a) Psychological nursing: nurses gave patients emotional comfort, encouraged patients to cooperate bravely, distracted patients’ attention, and conveyed the information that PCI was not painful to patients through verbal cues between medical staff. (b) Strengthening the prevention of intraoperative complications: nurses observed whether there was skin ecchymosis or peripheral blood circulation disorder, once found, nurses promptly notified the doctor and cooperated with the doctor for treatment. Because patients took a large number of drugs before surgery, PCI surgery may stimulate the vagus nerve of the patient’s heart to some extent during the operation, resulting in vomiting symptoms during the operation. Nurses paid close attention to the patient’s condition during the operation. Once vomiting occurred, nurses asked the patient to turn the head to one side, kept the body still, and cleaned up the vomit.
3. Postoperative nursing. The observation and preventive intervention of postoperative complications were strengthened, including: (a) Puncture site nursing: After PCI, a sandbag with appropriate weight was placed at the puncture site for compression, the limb on the puncture side was immobilization within 5 h after surgery, and the head of the patient’s bed was raised by 20–30°. Nurses assessed the temperature of the skin around the piercing. If the skin temperature rose, nurses were alert for infection at the piercing site. (b) Prevention of hypotension: Nurses explained to the patient before removing the sheath to reduce the tension of patients. Nurses gently reduced the pull on the blood vessel as much as possible during the operation, avoided excessive pressure, and prepared the related drugs to raise blood pressure. The patient’s blood pressure was measured regularly. (c) Thromboembolism prevention: Nurses closely observed the pulsation of the radial artery and dorsal foot artery of both lower limbs, skin color and temperature, etc. Patients were instructed to get out of bed early after PCI (24 h after surgery). (d) Prevention of gastrointestinal bleeding: Patients were instructed to strengthen dietary guidance and avoid eating irritating, hard and other foods. The nurse asked the patient to observe the color of the stool.

## Observation indicators

1. The left ventricular ejection fraction (LVEF) as well as blood pressure was compared between 2 groups.
2. The time of lying in bed and hospital stay were recorded in both groups.
3. According to the self-rating anxiety scale (SAS) as well as self-rating depression scale (SDS) (21), the psychological state of patients was evaluated. The lower the score of SAS and SDS, the better the psychological state of the patients.
4. The Comprehensive Quality of Life Assessment Questionnaire (GQOLI-74) was adopted to measure the quality of life of 2 groups (22). This scale consisted of 20 factors and 4 dimensions. The lowest score for each dimension was 0, and the highest score is 100. The low score indicated the low quality of life.
5. The General Self-efficacy Scale (GSES) could be adopted to evaluate the self-efficacy of patients (23). According to the actual situation of patients, the items were selected as completely incorrect (1 point), somewhat correct (2 points), mostly correct (3 points), and completely correct (4 points). The higher the score, the higher the self-efficacy was.
6. The incidence of postoperative adverse reactions (deep vein thrombosis, hypothermia, abdominal distension, insomnia and myocardial infarction) in 2 groups was observed.
7. The nursing satisfaction questionnaire survey scale made by the hospital was adopted for assessing nursing satisfaction of patients, including nurse–patient communication, service skills, nursing knowledge, and health education. The highest score was 100, the lowest score was 0, ≥80 indicated satisfaction, <60 indicated dissatisfaction, and others indicated general satisfaction. Total satisfaction rate (%) = (number of satisfied cases + number of generally satisfied cases) ÷ Total number of cases ×100%.
8. Intestinal microbiome status: A disposable sterile cotton swab was used to swab stool samples from patients, the samples were stored in a low temperature environment for testing. 4.5 mL dilution was added to the samples for full shock during testing, and then the samples were continuously diluted to 1,000 mL by 10-fold dilution method. Lactobacillus, bifidobacterium and escherichia coli were inoculated, cultured, and isolated, and the number of bacteria was calculated.
9. 5 mL of fasting elbow venous blood was collected from all patients in the early morning after admission, centrifuged at 3,500 rpm for 15 min, and the upper serum was taken and stored at low temperature to be measured. The levels of hemoglobin (Hb), albumin (ALB), transferrin (TRF), prealbumin (PA) were examined.
10. Blood lipid levels including triglyceride (TG) level, low density lipoprotein cholesterol (LDL⁃C) content, high density lipoprotein cholesterol (HDL⁃C) level and total cholesterol (TC) level of the patients were measured.

## Statistical analysis

SPSS 24.0 statistical software was adopted for data analysis. Measurement data were expressed as (*x* ± *s*), and *t*-test was adopted for comparison. Count data were expressed as (*n*, %), and Fisher’s exact test was used for comparison. *p* < 0.05 meant statistical significance.

## Results

### LVEF and blood pressure in 2 groups

**Figure 1:**
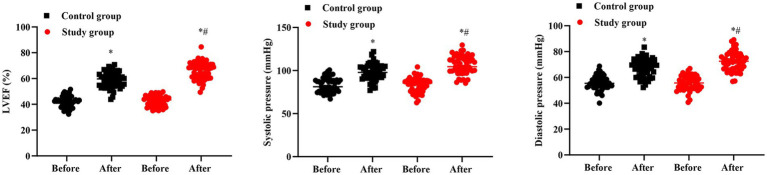
LVEF and blood pressure in 2 groups. ^#^*p* < 0.05, in contrast to before intervention, ^*^p < 0.05, in contrast to control group.

No significant difference was seen in LVEF and blood pressure between 2 groups prior to intervention (*p* > 0.05). Followed by intervention, the LVEF and blood pressure were elevated in 2 groups, and those in the SG presented higher than the CG (*p* < 0.05, Figure 1).

### Time of lying in bed and hospital stay in 2 groups

**Figure 2:**
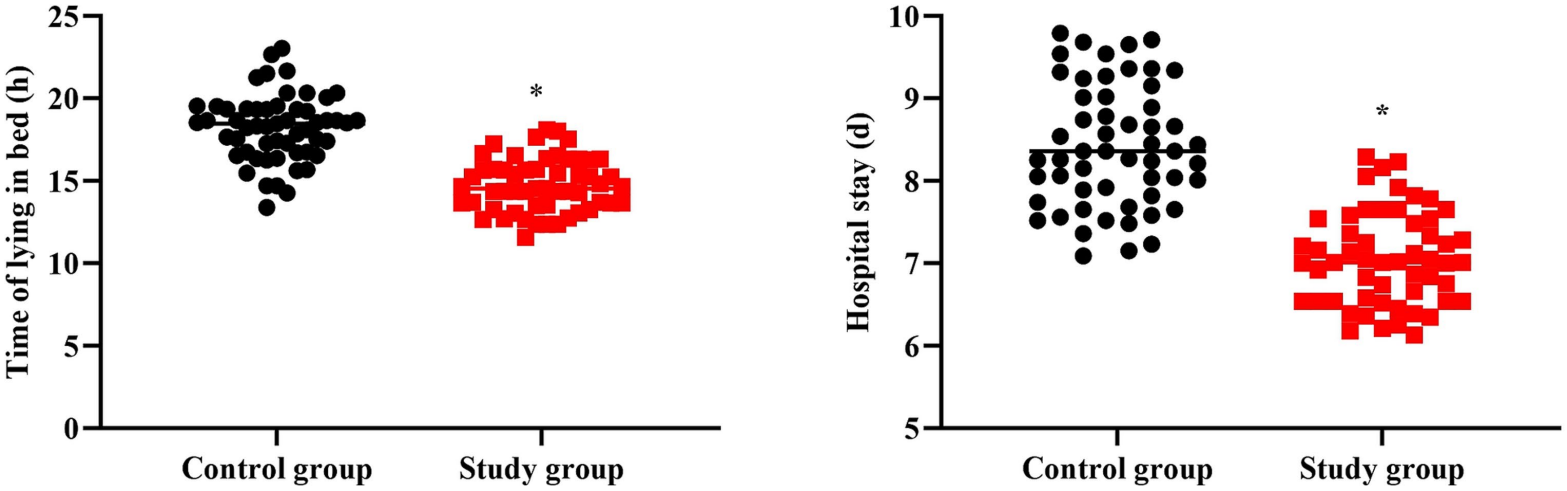
Time of lying in bed and hospital stay in 2 groups. ^*^p < 0.05.

In contrast to the CG, the time of lying in bed and hospital stay in the SG were shorter (*p* < 0.05, Figure 2).

### Negative emotions in 2 groups

**Figure 3:**
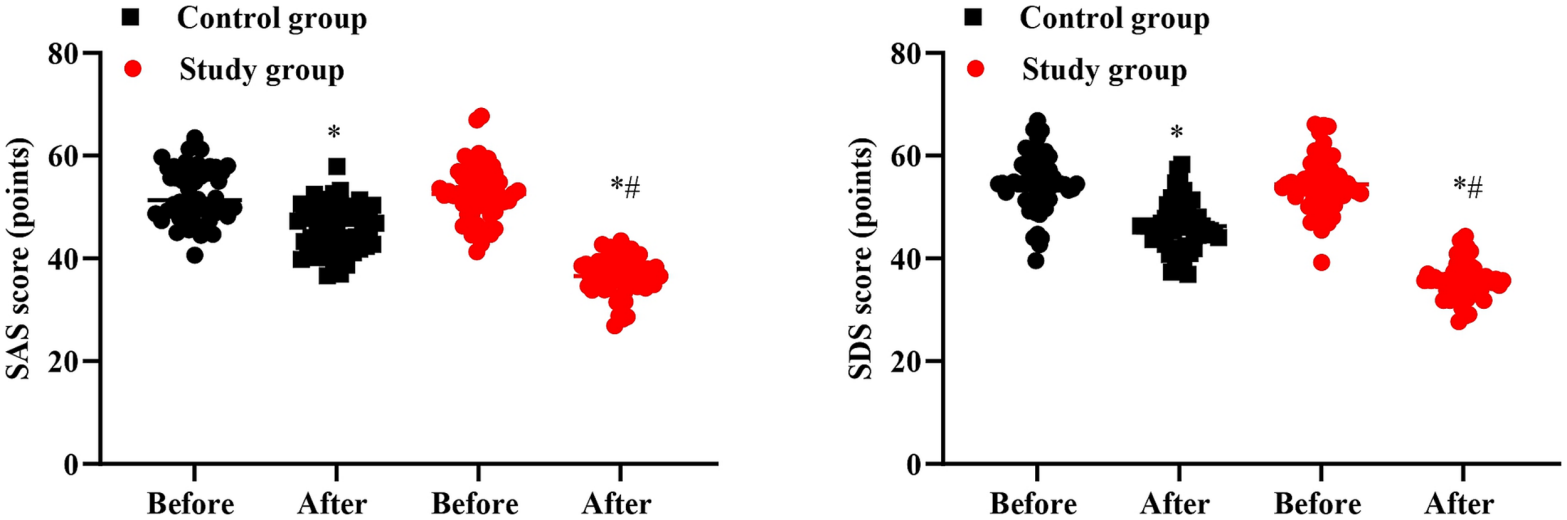
Negative emotions in 2 groups. ^#^*p* < 0.05, in contrast to before intervention, ^*^p < 0.05, in contrast to control group.

No significant difference was seen in SAS and SDS scores between 2 groups prior to intervention (*p* > 0.05). Followed by intervention, the SAS and SDS scores were declined in 2 groups, and those in the SG presented reduction than the CG (*p* < 0.05, Figure 3).

### Quality of life in 2 groups

**Figure 4:**
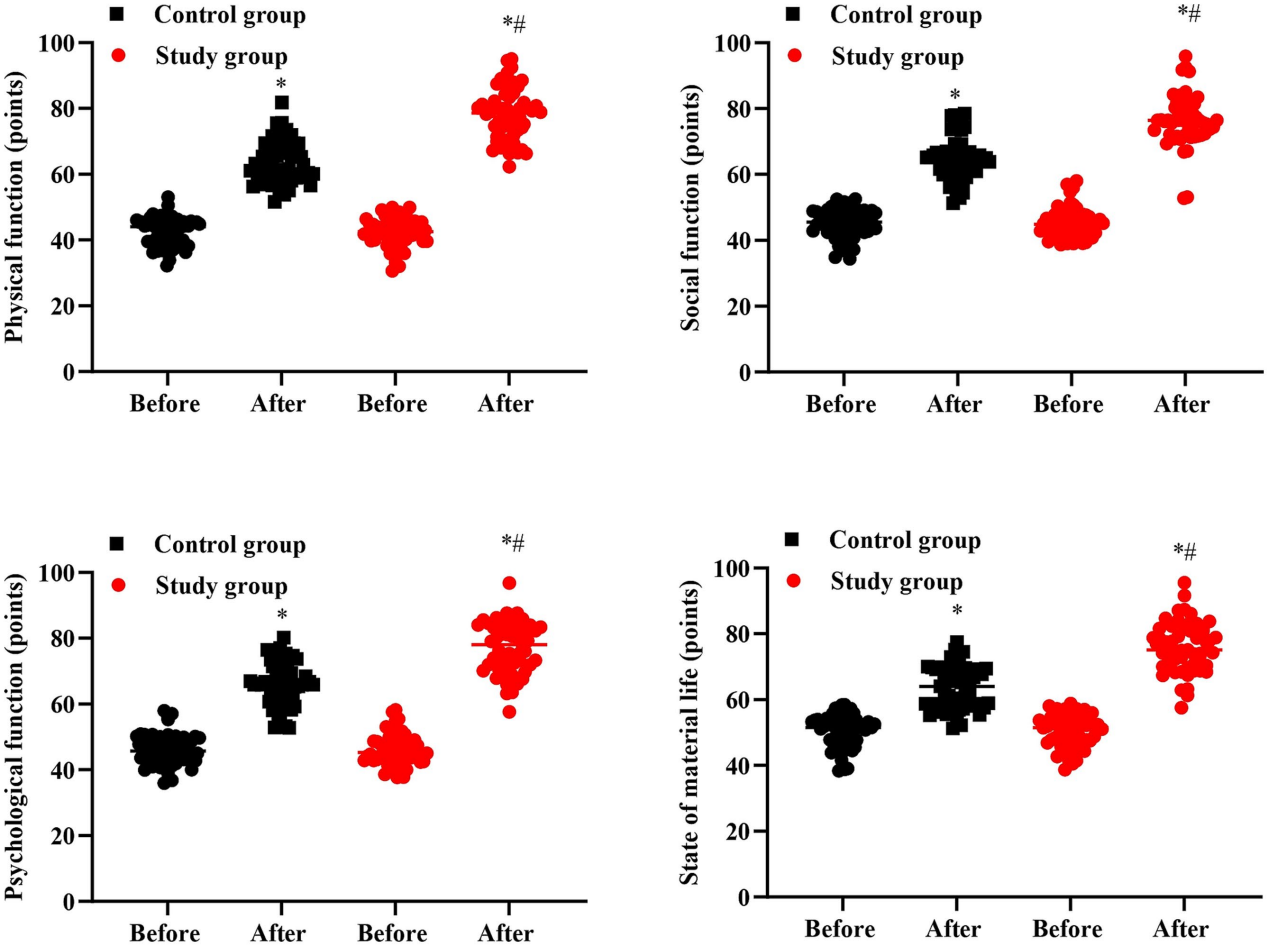
Quality of life in 2 groups. ^#^*p* < 0.05, in contrast to before intervention, ^*^p < 0.05, in contrast to control group.

No significant difference was seen in GQOLI-74 scores between 2 groups prior to intervention (*p* > 0.05). Followed by intervention, the GQOLI-74 scores were elevated in 2 groups, and those in the SG presented elevation than the CG (*p* < 0.05, Figure 4).

### Self-efficacy in 2 groups

**Figure 5:**
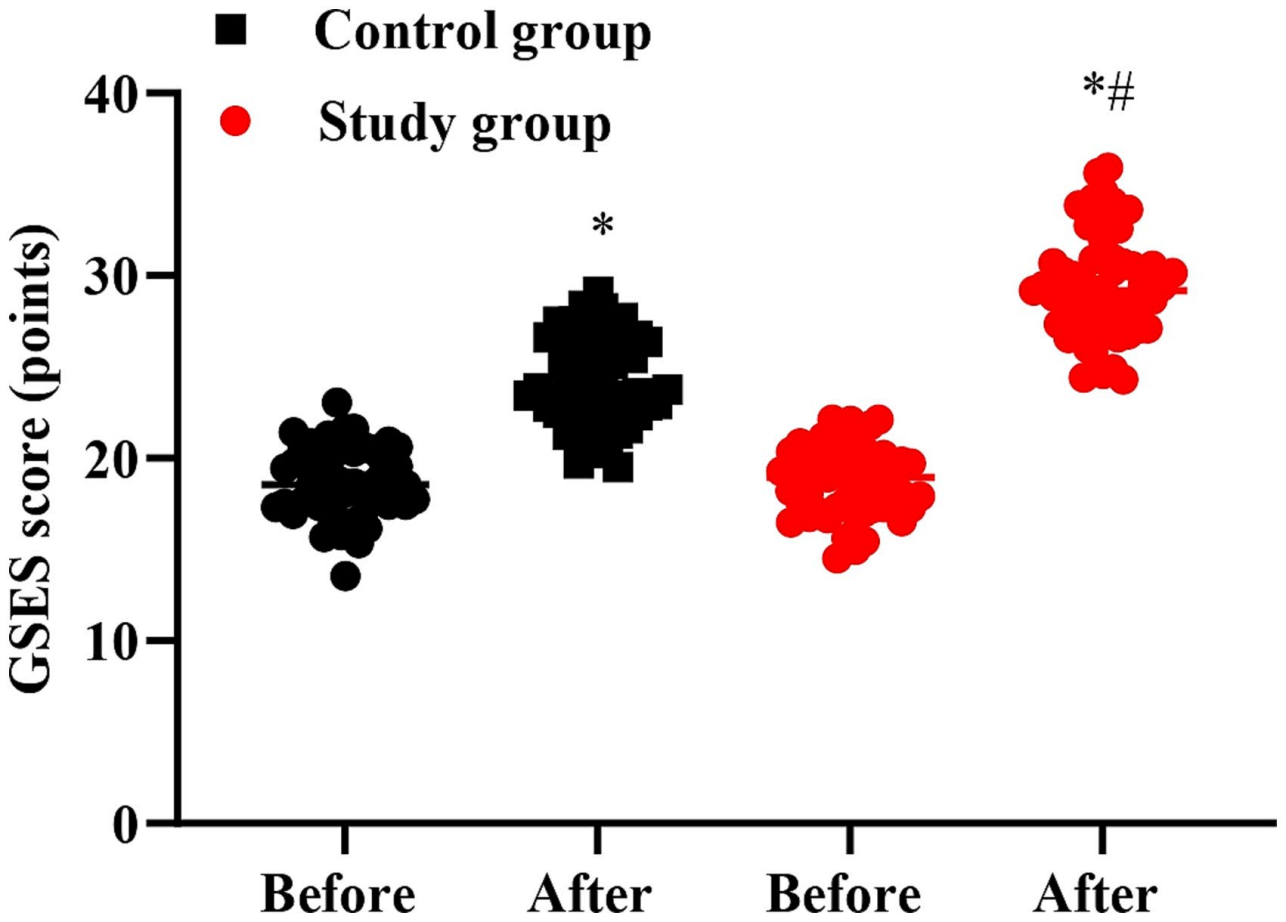
Self-efficacy in 2 groups. ^#^*p* < 0.05, in contrast to before intervention, ^*^p < 0.05, in contrast to control group.

No significant difference was seen in GSES score between 2 groups prior to intervention (*p* > 0.05). Followed by intervention, the GSES score was elevated in 2 groups, and that in the SG presented elevation than the CG (*p* < 0.05, Figure 5).

### Incidence of complications in 2 groups

In contrast to the CG, the incidence of complications in the SG presented lower (*p* < 0.05, Table 1).

**Table 1:** Incidence of complications in 2 groups.

Groups	*N*	Deep vein thrombosis	Hypothermia	Abdominal distension	Insomnia	Myocardial infarction	Total incidence rate
Control group	55	1	2	3	2	2	10 (18.18%)
Study group	55	0	0	0	1	1	2 (3.64%)
*P*							0.028

### Nursing satisfaction in 2 groups

Table 2 displayed that in contrast to the CG, the nursing satisfaction of patients in the SG presented better (*p* < 0.05).

**Table 2:** Nursing satisfaction in 2 groups.

Groups	*N*	Satisfied	Generally satisfied	Dissatisfied	Total satisfaction rate
Control group	55	25	22	8	47 (85.45%)
Study group	55	28	26	1	54 (98.18%)
*P*					0.031

### Number of intestinal flora in 2 groups

**Figure 6:**
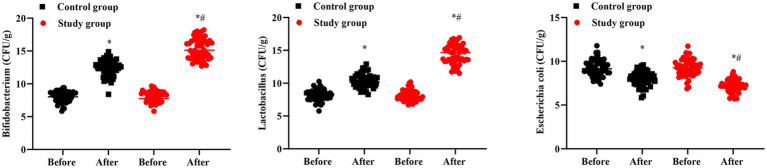
Number of intestinal flora in 2 groups. ^#^*p* < 0.05, in contrast to before intervention, ^*^p < 0.05, in contrast to control group.

No significant difference was seen in number of lactobacillus, bifidobacterium and escherichia coli between 2 groups prior to intervention (*p* > 0.05). After intervention, the number of lactobacillus and bifidobacterium was elevated while the number of escherichia coli was declined in 2 groups, and the improvements of lactobacillus, bifidobacterium and escherichia coli in the SG were more significant than the CG (*p* < 0.05, Figure 6).

### Nutritional status in 2 groups

**Figure 7:**
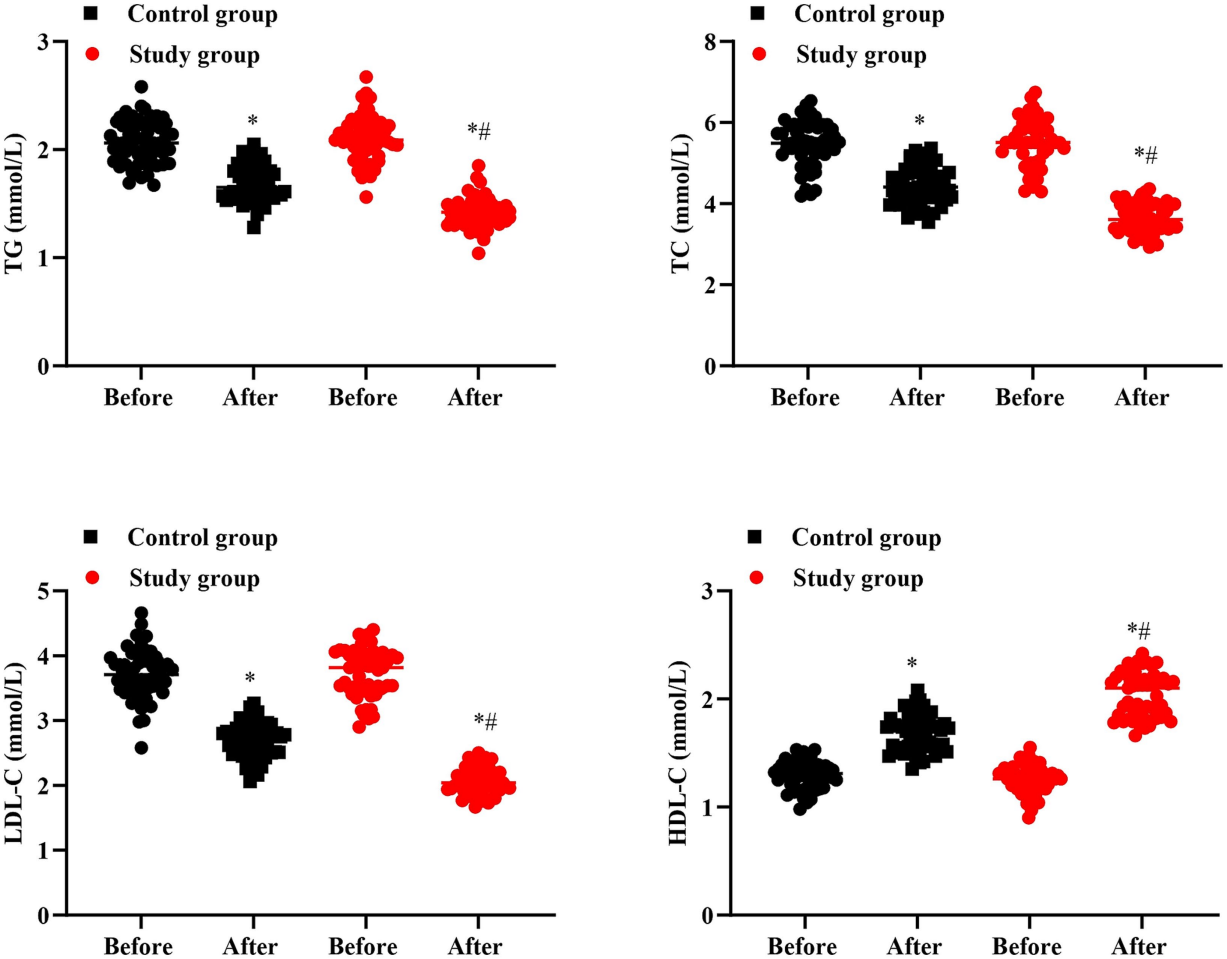
Nutritional status in 2 groups. ^#^*p* < 0.05, in contrast to before intervention, ^*^p < 0.05, in contrast to control group.

No significant difference was seen in Hb, ALB, TRF, and PA levels between 2 groups prior to intervention (*p* > 0.05). After intervention, Hb, ALB, TRF, and PA levels were elevated in 2 groups, and those in the SG were higher than the CG (*p* < 0.05, Figure 7).

### Blood lipid levels in 2 groups

**Figure 8:**
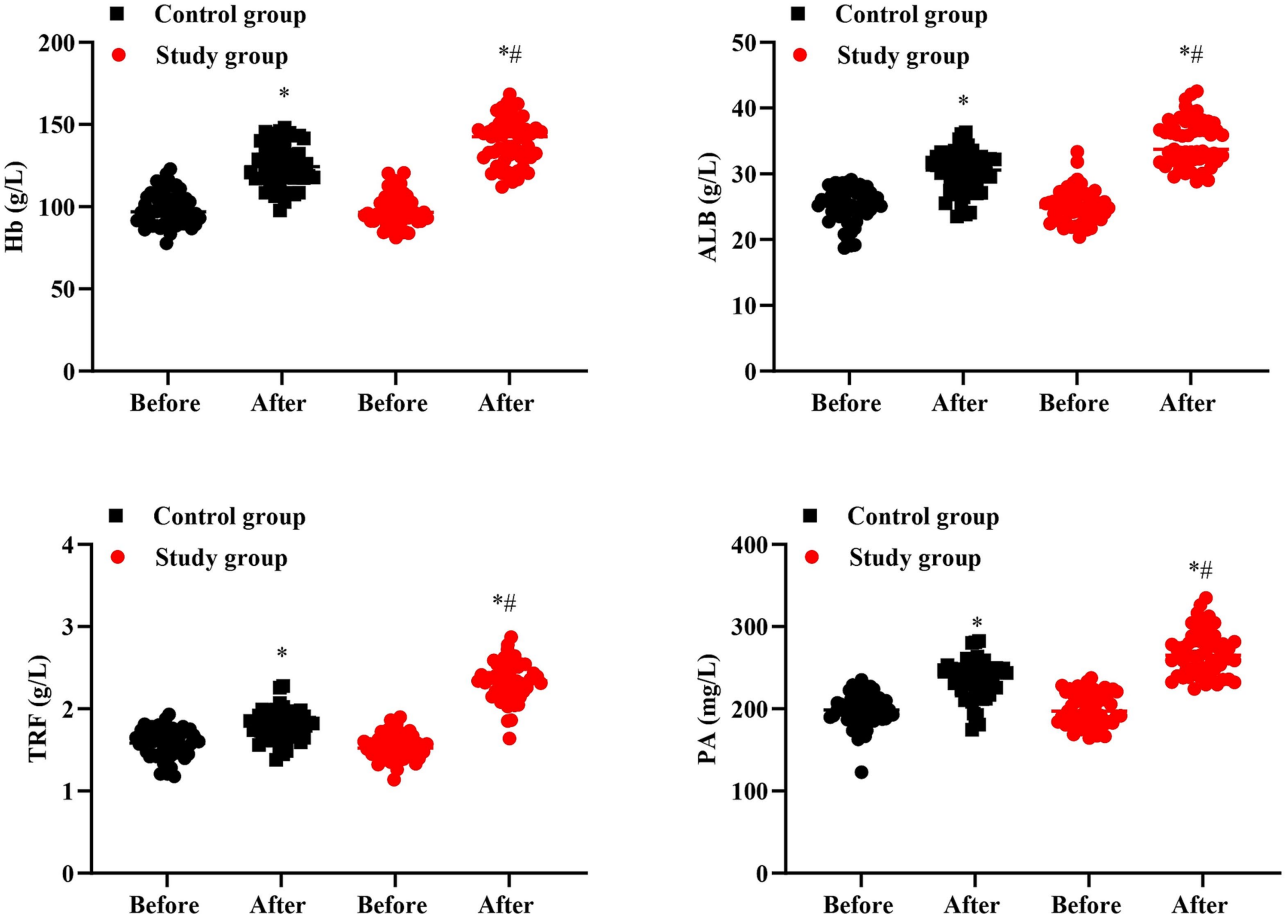
Blood lipid levels in 2 groups. ^#^*p* < 0.05, in contrast to before intervention, ^*^p < 0.05, in contrast to control group.

No significant difference was seen in TG, TC, LDL-C and HDL-C levels between 2 groups prior to intervention (*p* > 0.05). After intervention, TG, TC and LDL-C levels were declined while HDL-C level was elevated in 2 groups, and the improvements of TG, TC, LDL-C and HDL-C in the SG were more significant than the CG (*p* < 0.05, Figure 8).

## Discussion

CHD is more common in middle-aged and elderly people (24). With the acceleration of the aging process of social population, the proportion of elderly CHD patients increases (25). Analyzing the causes of the disease, it is closely related to the patients’ bad living habits, such as high sugar, high fat diet, long stay up late, smoking, alcoholism and so on (26). PCI is the main method to treat this disease, which can effectively relieve patients’ angina pectoris and improve myocardial blood perfusion (27). However, patients with improper emotional control after surgery will elevate the risk of cardiovascular adverse events along with reduce the quality of life of patients (28). Therefore, how to carry out effective nursing is the key to ensure the effect of PCI surgery and the safety of patients.

The purpose of emergency comprehensive nursing is to obtain better quality of life for patients while treating, so that medical resources can be used reasonably and fully (29). The orderly development of emergency comprehensive nursing requires professional skills, good communication skills and a high sense of responsibility of nursing staff, putting the needs of patients in the first place, and increasing the comfort of patients during hospitalization (30). The connotation of emergency comprehensive nursing is the overall responsibility system of nursing staff, ensuring the safety and comfort of patients, and improving the professional value of nursing staff (31). Previous studies have revealed that emergency comprehensive nursing has more advantages in relieving patients’ negative emotions and speeding up the rehabilitation process than conventional nursing (32).

The main pathological feature of CHD is atherosclerosis, in which metabolic abnormalities and inflammatory reactions play an important role, among which intestinal flora is a new target discovered in recent years (33). Studies have found that intestinal flora controls the absorption and metabolism of nutrients through proteolytic pathways and glycolysis pathways, and participates in the occurrence and development of cardiovascular, cerebrovascular, kidney and other diseases (34). Other studies have confirmed through animal experiments that the number of lactobacillus increases and the number of bacteroides decreases in the CHD group, and the progression of CHD can be delayed after the mice in the CHD group are administrated with bacteroides (35).

Bifid triple viable capsules dissolving at intestines are a combination of bifidobacterium longiformis, *lactobacillus acidophilus*, and enterococcus faecalis (36). Lactobacillus and bifidobacterium are microorganisms in the body that are beneficial to host health, which can improve intestinal function and regulate immune function (37). At the same time, they have biological barrier and nutritional effects on human health. After intestinal flora is disturbed, the metabolic components of bile acids in the body are changed, and the induced dyslipidemia can promote atherosclerosis and then increase the risk of major adverse cardiovascular events (38). Lactobacillus and bifidobacterium can produce a hydrolase combining bile acid, which can promote the liver to synthesize bile acid with cholesterol as raw material, accelerate the speed of cholesterol transformation, and achieve the effect of lipid lowering (39). When the blood lipid gradually improves to the normal state, the internal environment of the body on which the intestinal microbial colonies depend tends to be suitable for the survival and colonization of beneficial bacteria, improve the growth, reproduction and metabolism level of beneficial bacteria, hinder the adhesion of pathogenic bacteria, improve the level of intestinal microbes, and reduce the occurrence of major adverse cardiovascular events (40).

In our study, the results indicated that after intervention, the LVEF and blood pressure in the SG presented higher than the CG, the time of lying in bed and hospital stay in the SG presented shorter than the CG, and the incidence of complications in the SG presented lower than the CG, suggesting that bifid triple viable capsules dissolving at intestines combined with emergency comprehensive nursing could reduce the impact of PCI on the cardiovascular function of patients, promote the postoperative recovery along with reduce the risk of complications of CHD patients, which was in accordance with former studies (41, 42).

Besides, our study indicated that after intervention, the SAS as well as SDS scores in the SG presented lower when comparing with the CG, the GQOLI-74 scores in the SG presented higher than the CG, the GSES score in the SG presented higher when comparing with the CG, and the nursing satisfaction in the SG presented higher when comparing with the CG. All these results implied that bifid triple viable capsules dissolving at intestines combined with emergency comprehensive nursing could reduce the negative emotions, promote the quality of life and self-efficacy along with elevate the nursing satisfaction of CHD patients. Consistently, Zhang et al. have indicated that comprehensive nursing can improve the pregnancy outcomes as well as has positive significance in reducing negative emotions of patients with polycystic ovary syndrome (43). Yang et al. have pointed that comprehensive intervention can elevate the life quality of elderly patients with Alzheimer Disease (44). Jalal Moludi et al. have indicated that probiotic supplementation in patients with PCI post-myocardial infarction has beneficial effects on depressive symptoms (45).

In addition, our study indicated that after intervention, the improvements of lactobacillus, bifidobacterium and escherichia coli in the SG were more significant than the CG, Hb, ALB, TRF, and PA levels were elevated in 2 groups, and those in the SG were higher than the CG, and the improvements of TG, TC, LDL-C and HDL-C in the SG were more significant than the CG, suggesting that bifid triple viable capsules dissolving at intestines combined with emergency comprehensive nursing could improve the intestinal microbiome status, enhance the nutritional status and improve the blood lipid levels of CHD patients after PCI, which was in line with previous studies (46, 47).

In conclusion, our study demonstrates that bifid triple viable capsules dissolving at intestines combined with emergency comprehensive nursing can improve the cardiovascular function, decrease the risk of complications, promote the self-efficacy and quality of life, improve the intestinal microorganism and nutritional status and improve the blood lipid levels of CHD patients after PCI.

## Conclusion

This study demonstrated that the combination of bifid triple viable capsules dissolving at intestines with emergency comprehensive nursing significantly improves cardiovascular function, shortens recovery time, enhances self-efficacy, and promotes the quality of life in coronary heart disease patients’ post-percutaneous coronary intervention. Additionally, the intervention effectively restores intestinal microbiota balance, optimizes nutritional status, and improves lipid profiles, offering a novel approach to enhancing the outcomes of PCI patients.

## Data Availability

The datasets presented in this study can be found in online repositories. The names of the repository/repositories and accession number(s) can be found in the article/supplementary material.
